# Supplementary material for: Analysis and modeling of cancer drug responses using cell cycle phase-specific rate effects
Source: Nat Commun. 2023 Jun 10;14:3450. doi: 10.1038/s41467-023-39122-z (PMC10257663; doi:10.1038/s41467-023-39122-z)
Supplement: Supplementary file 1 — Supplementary Information [file 41467_2023_39122_MOESM1_ESM.pdf]

Analysis and modeling of cancer drug responses using cell cycle phase-specific rate effects

Supplementary Information

Sean M. Gross[1,†], Farnaz Mohammadi[2,†], Crystal Sanchez-Aguila[1], Paulina J. Zhan[1], Tiera A. Liby[1], Mark A. Dane[1], Aaron S. Meyer[2,^], and Laura M. Heiser[1,^,*]

[1]Department of Biomedical Engineering, Knight Cancer Institute, Oregon Health & Science University, Portland, OR

[2]Department of Bioengineering, University of California, Los Angeles; Jonsson Comprehensive Cancer Center, University of California at Los Angeles, Los Angeles, CA

[†]These authors contributed equally

^These authors jointly supervised this work

Supp. Figure 1

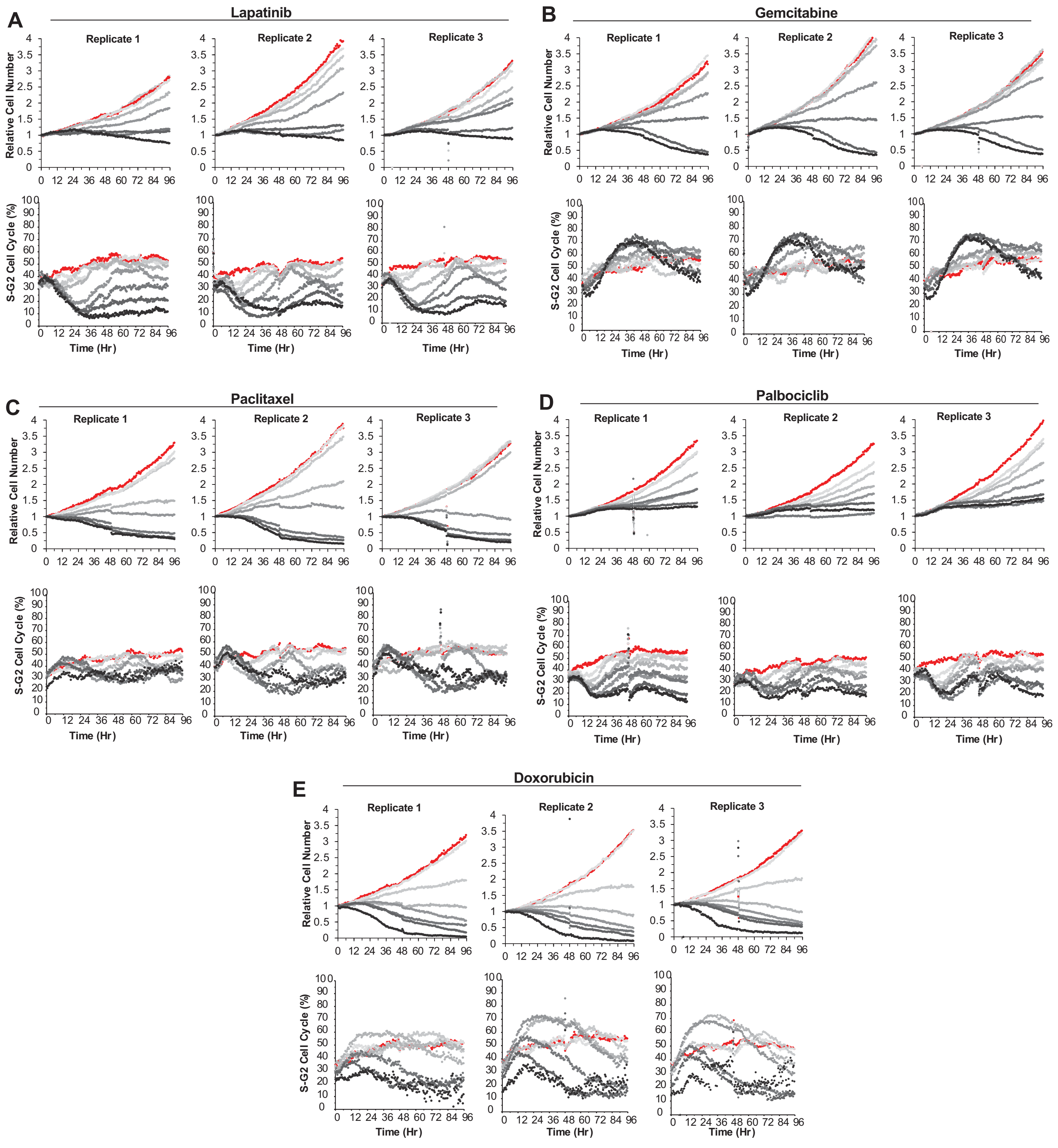

Supplementary Figure 1. Individual replicates for AU565 drug responses show similar temporal dynamics and drug-induced changes to cell cycle. Panels show relative cell numbers and S-G_2 normalized cell numbers for lapatinib (**A**), gemcitabine (**B**), paclitaxel (**C**), palbociclib (**D**), and doxorubicin (**E**) treatments for three biological replicates. Five drug concentrations (gray lines) and untreated control (red line) are plotted. Source data are provided on Synapse..

Supp. Figure 2

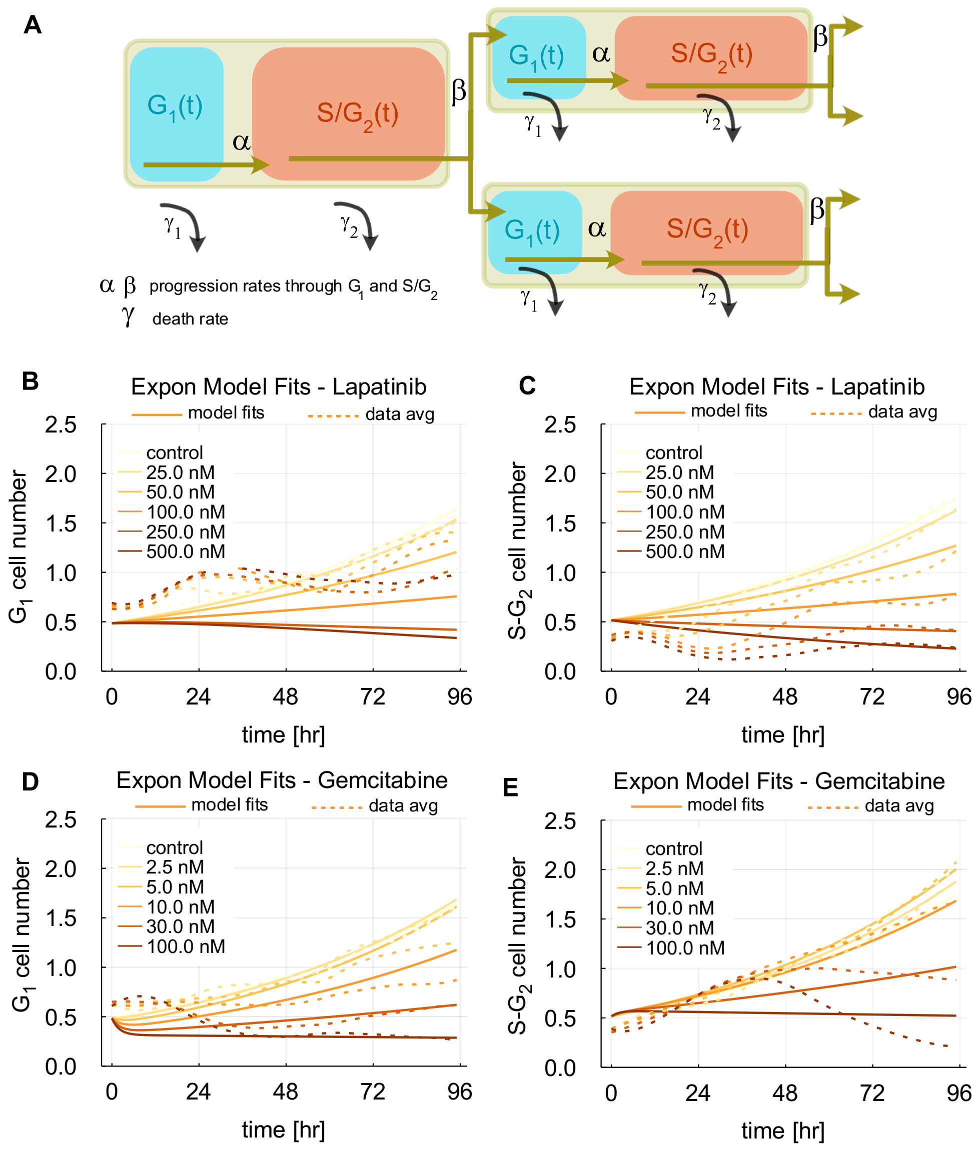

Supplementary Figure 2. An exponential cell cycle model without incorporating delay times fails to capture the dynamics of drug response. A. The transition diagram for a simple dynamical model with 2 phases (G_1 and S-G_2) and without the LCT. α and β, are the transition rates from G_1 to S-G_2 and vice versa, γ_1 and γ_2 are the death rates in G_1 and S-G_2, respectively. **B-E.** Exponential cell cycle model simulations of G_1 and S-G_2 cell numbers over time for control and 5 concentrations of lapatinib (**B-C**) and gemcitabine (**D-E**) (solid lines), respectively, overlayed with the average of three experimental replicates (dashed lines). Source data are provided on Synapse.

Supp Figure 3

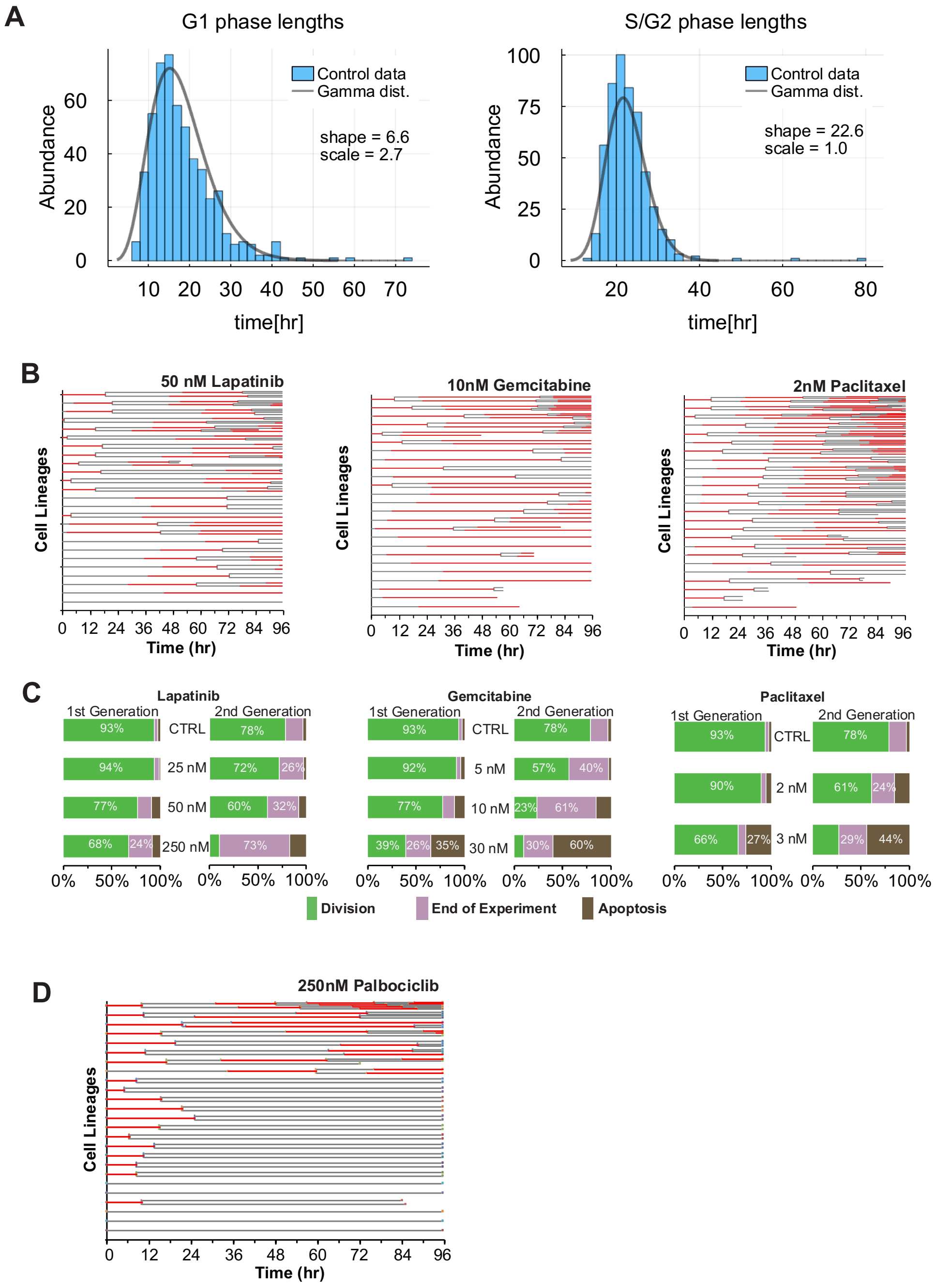

Supplementary Figure 3. Analysis of single cell tracking data reveals drug-specific cell cycle phase effects in AU565 cells. A. Lineage trees of 25 lineages across 96H for various drug treatments. Tracks are colored coded based on cell cycle phase: gray indicates G_1 and red indicates $S\text{-}G_2$ phase. Track splitting indicates mitosis, and track ending prior to 96H corresponds to apoptosis. **B.** Quantification of cell outcomes (division, cell death, viable at end of experiment) for cells from the first and second generations treated with lapatinib, gemcitabine, or paclitaxel. **C.** Gamma distribution of G_1 and $S\text{-}G_2$ phase durations for cells in control condition with sample size of 520 and 514 for G_1 and $S\text{-}G_2$ phases, respectively. **D.** Lineage trees for 25 lineages across 96H after treatment with Palbociclib. Source data are provided on Synapse.

Supp. Figure 4

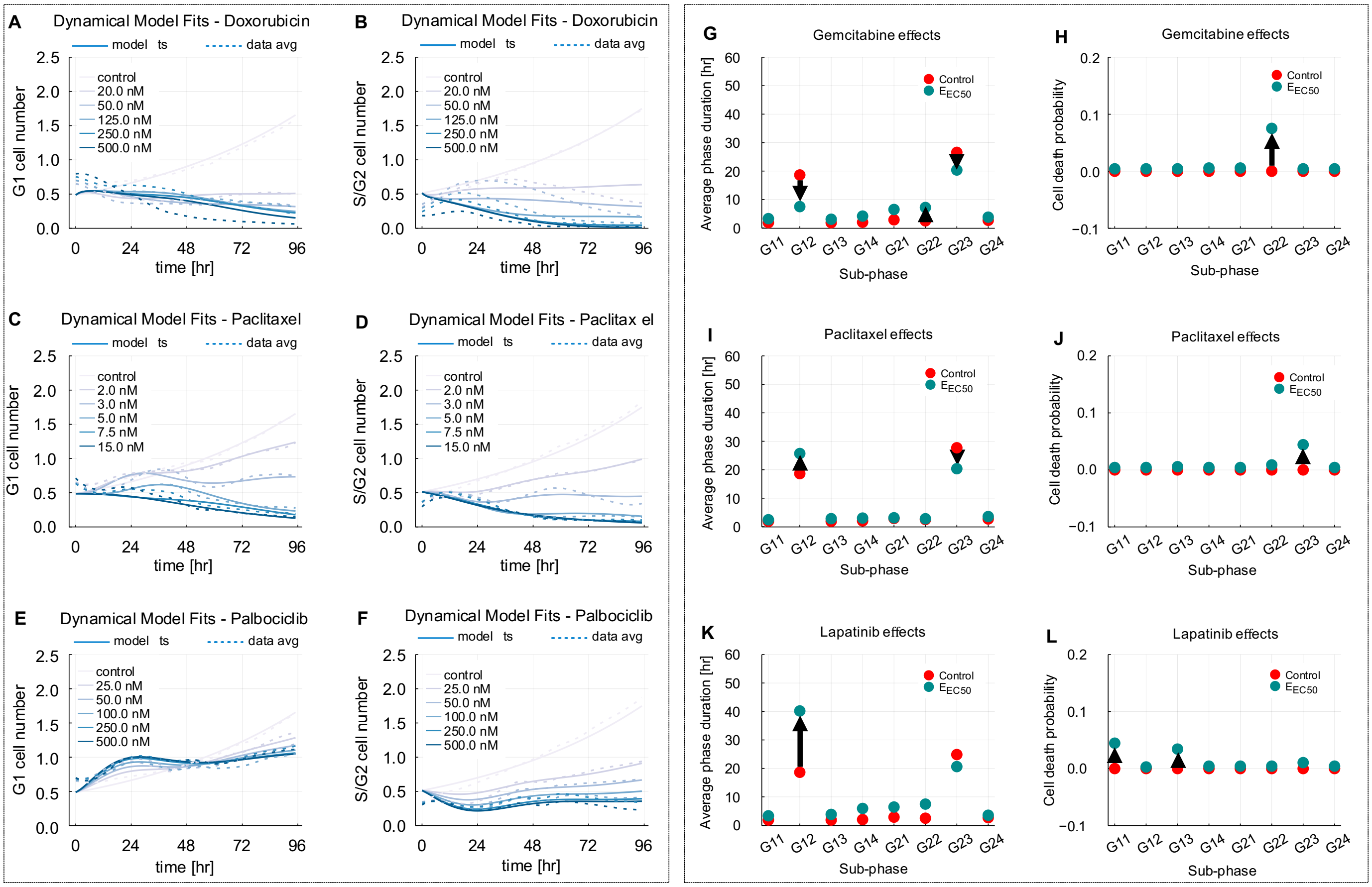

Supplementary Figure 4. A dynamical model of the cell cycle captures the dynamics of drug response. A-F. G_1 and S-G_2 cell numbers overtime, respectively, for the control and treatment at 5 concentrations (solid lines) for doxorubicin (**A-B**) paclitaxel (**C-D**), and palbociclib (**E-F**) overlayed with the average of three corresponding experimental replicates (dashed lines). **G-L**. The average phase durations in G_1 and S-G_2 phases for selected drug treatments. The arrow shows the shift from the control condition to the drug effect at the half maximum concentration (E_{EC50}). Source data are provided on Synapse.

Supp. Figure 5

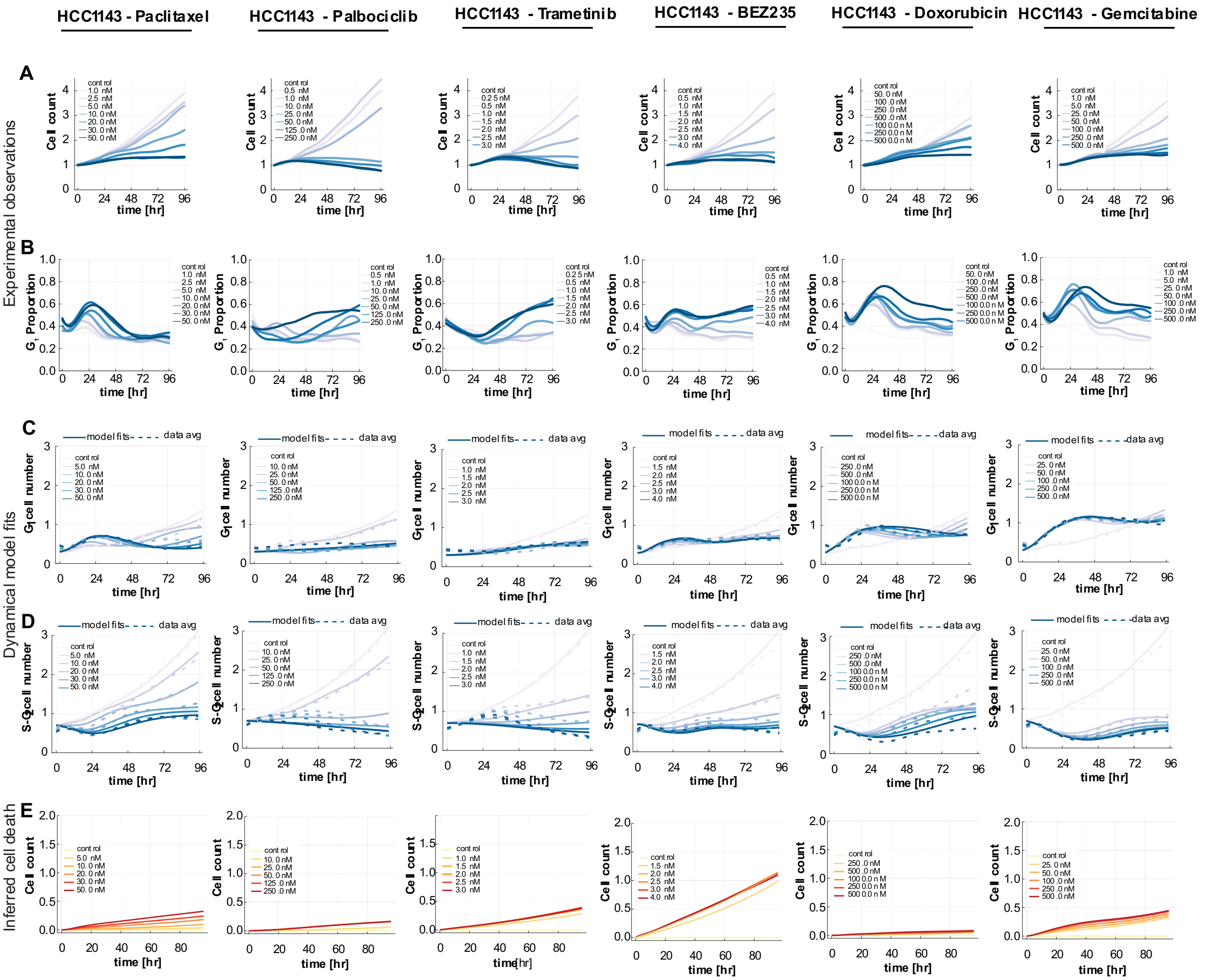

Supplementary Figure 5. The introduced dynamical model captures the cell cycle dynamics of drug response in TNBC cell line HCC1143. A, B. Experimentally observed drug-induced changes to cell numbers (**A**) and G_1 cell cycle phase proportion (**B**) after dose-escalation treatment with a panel of inhibitors. **C,D.** G_1 and S-G_2 fits overtime, respectively, for the untreated and treatment at 5 concentrations (solid lines) overlayed with the average of three corresponding experimental replicates (dashed lines) for 6 drug treatments. **E.** Inferred accumulated dead cells over time for 6 drug treatments. Source data are provided on Synapse.

Supp. Figure 6

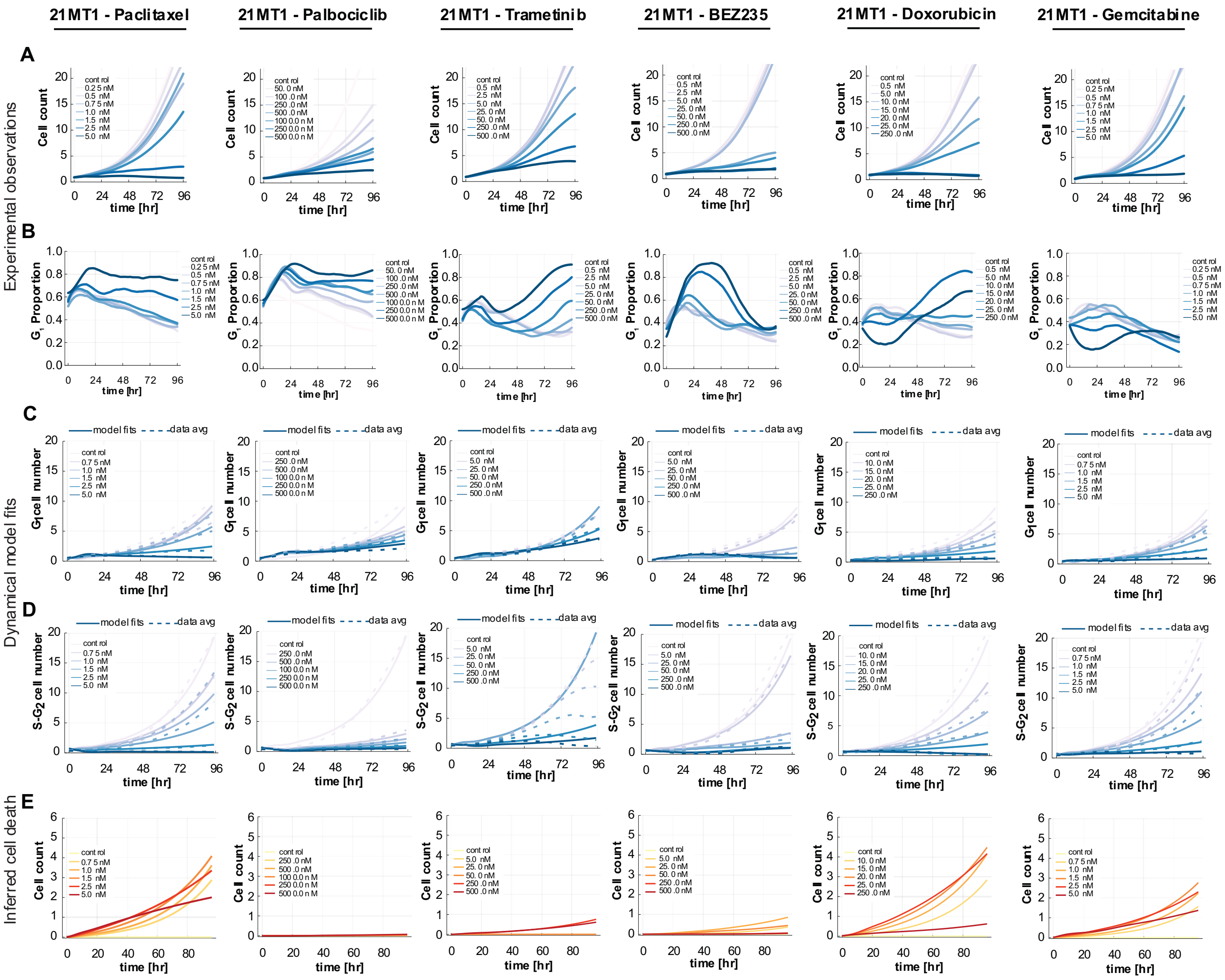

Supplementary Figure 6. The introduced dynamical model captures the cell cycle dynamics of drug response in 21MT1 cell line. A, B. Experimentally observed drug-induced changes to cell numbers (**A**) and G_1 cell cycle phase proportion (**B**) after dose-escalation treatment with a panel of inhibitors. **C,D.** G_1 and S-G_2 fits overtime, respectively, for the untreated and treatment at 5 concentrations (solid lines) overlayed with the average of three corresponding experimental replicates (dashed lines) for 6 drug treatments. **E.** Inferred accumulated dead cells over time for 6 drug treatments. Source data are provided on Synapse.

Supp. Figure 7

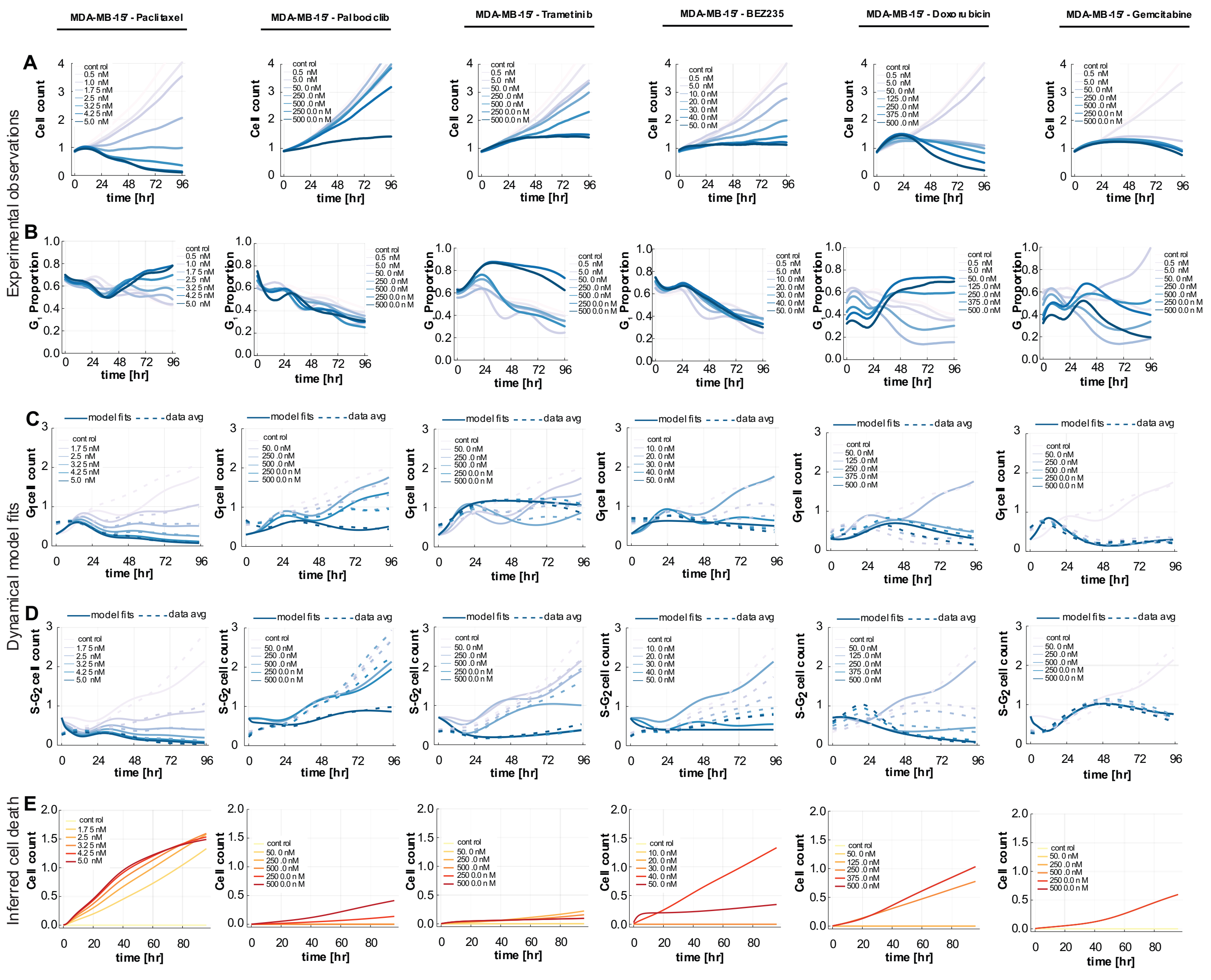

Supplementary Figure 7. The introduced dynamical model captures the cell cycle dynamics of drug response in TNBC cell line MDAMB175. A,B. Experimentally observed drug-induced changes to cell numbers (**A**) and G_1 cell cycle phase proportion (**B**) after dose-escalation treatment with a panel of inhibitors. **C,D.** G_1 and S-G_2 fits overtime, respectively, for the untreated and treatment at 5 concentrations (solid lines) overlayed with the average of three corresponding experimental replicates (dashed lines) for 6 drug treatments. **E.** Inferred accumulated dead cells over time for 6 drug treatments. Source data are provided on Synapse.

Supp. Figure 8

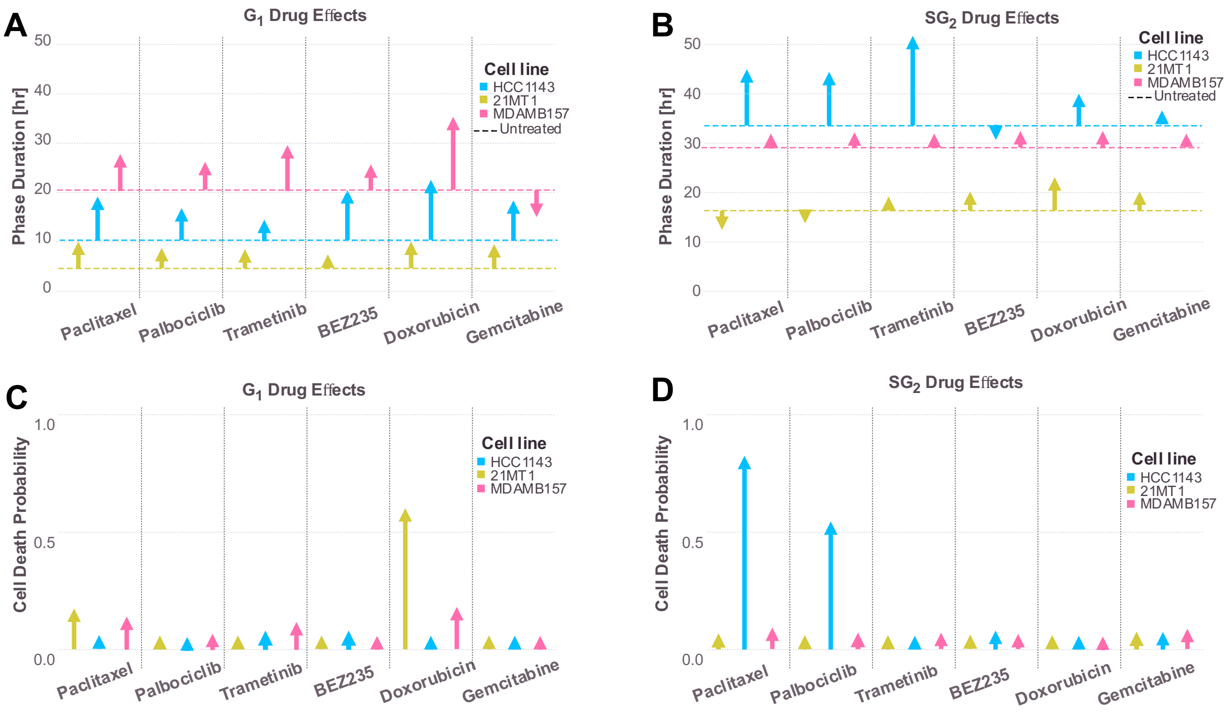

Supplementary Figure 8. Summary of inferred cell cycle drug effects at half maximum concentration compared to untreated. A-B. The average phase durations in G_1 **(A)** and S-G_2 **(B)** phases for HCC1143 (blue), 21MT1 (olive) and MDA-MB-157 (pink) treated with paclitaxel, palbociclib, trametinib, BEZ235, doxorubicin, and gemcitabine. The dashed lines show the average phase duration at untreated for each cell line. **C-D**. The cell death probability in G_1 **(C)** and S-G_2 **(D)** phases for HCC1143 (blue), 21MT1 (olive) and MDA-MB-157 (pink) treated with the same panel of drugs. The arrows show the quantity of increase or decrease in the effects from untreated to the half maximal concentration (E_{EC50}). Source data are provided on Synapse.

Supplementary Tables

Symbol	Units	Explanation
α_1	1/cell/hr	Cells progression rate through the first quarter of G1.
α_2	1/cell/hr	Cells progression rate through the second quarter of G1.
α_3	1/cell/hr	Cells progression rate through the third quarter of G1.
α_4	1/cell/hr	Cells progression rate through the fourth quarter of G1.
β_1	1/cell/hr	Cells progression rate through the first quarter of S/G2.
β_2	1/cell/hr	Cells progression rate through the second quarter of S/G2.
β_3	1/cell/hr	Cells progression rate through the third quarter of S/G2.
β_4	1/cell/hr	Cells progression rate through the fourth quarter of S/G2.
γ_{11}	1/cell/hr	Cells rate of death within the first quarter of G1.
γ_{12}	1/cell/hr	Cells rate of death within the second quarter of G1.
γ_{13}	1/cell/hr	Cells rate of death within the third quarter of G1.
γ_{14}	1/cell/hr	Cells rate of death within the fourth quarter of G1.
γ_{21}	1/cell/hr	Cells rate of death within the first quarter of S/G2.
γ_{22}	1/cell/hr	Cells rate of death within the second quarter of S/G2.
γ_{23}	1/cell/hr	Cells rate of death within the third quarter of S/G2.
γ_{24}	1/cell/hr	Cells rate of death within the fourth quarter of S/G2.

Supplementary Table 1. Explanation of the rate parameters within the ODE model.

Cell line	Drug	EC50 concentration [nM]
HCC1143	Paclitaxel	1.46
HCC1143	Palbociclib	86.46
HCC1143	Trametinib	3.71
HCC1143	BEZ235	34.94
HCC1143	Doxorubicin	159.16
HCC1143	Gemcitabine	0.74
21MT1	Paclitaxel	1.55
21MT1	Palbociclib	19.1
21MT1	Trametinib	220.8
21MT1	BEZ235	12.52
21MT1	Doxorubicin	51.68
21MT1	Gemcitabine	1.51
MDAMB157	Paclitaxel	2.01
MDAMB157	Palbociclib	3594.01
MDAMB157	Trametinib	658.20
MDAMB157	BEZ235	10.90
MDAMB157	Doxorubicin	247.58
MDAMB157	Gemcitabine	0.64

Supplementary Table 2. The concentration at half maximal effect for each cell line and treatment.
